# Adaptation of IoT with Blockchain in Food Supply Chain Management: An Analysis-Based Review in Development, Benefits and Potential Applications

**DOI:** 10.3390/s22218174

**Published:** 2022-10-25

**Authors:** Amanpreet Kaur, Gurpreet Singh, Vinay Kukreja, Sparsh Sharma, Saurabh Singh, Byungun Yoon

**Affiliations:** 1 Chitkara University Institute of Engineering & Technology, Chitkara University, Rajpura 173212, Punjab, India; 2 Department of Computer Science & Engineering, Punjab Institute of Technology, Rajpura 140401, Punjab, India; 3 Department of Computer Science and Engineering, National Institute of Technology, Srinagar 190001, Jammu and Kashmir, India; 4 Department of Industrial and Systems Engineering, Dongguk University, Seoul 04620, Korea

**Keywords:** blockchain, food supply chain, IoT, cloud computing

## Abstract

In today’s scenario, blockchain technology is an emerging area and promising technology in the field of the food supply chain industry (FSCI). A literature survey comprising an analytical review of blockchain technology with the Internet of things (IoT) for food supply chain management (FSCM) is presented to better understand the associated research benefits, issues, and challenges. At present, with the concept of farm-to-fork gaining increasing popularity, food safety and quality certification are of critical concern. Blockchain technology provides the traceability of food supply from the source, i.e., the seeding factories, to the customer’s table. The main idea of this paper is to identify blockchain technology with the Internet of things (IoT) devices to investigate the food conditions and various issues faced by transporters while supplying fresh food. Blockchain provides applications such as smart contracts to monitor, observe, and manage all transactions and communications among stakeholders. IoT technology provides approaches for verifying all transactions; these transactions are recorded and then stored in a centralized database system. Thus, IoT enables a safe and cost-effective FSCM system for stakeholders. In this paper, we contribute to the awareness of blockchain applications that are relevant to the food supply chain (FSC), and we present an analysis of the literature on relevant blockchain applications which has been conducted concerning various parameters. The observations in the present survey are also relevant to the application of blockchain technology with IoT in other areas.

## 1. Introduction

The growth of agricultural crops and the management of logistics in food must be stringently monitored. The supply chain of agricultural products and crops is a critical aspect related to product safety; the risk of food spoilage and potential poisoning has led to the increased focus on traceability enhancement. Agricultural food and products are particularly vulnerable, and consumers are quite concerned about the quality, nutritive value, and safety of the food they consume. In fact, the food crisis was ranked as the seventh-highest risk in the year 2018 by the World Economic Forum [1]. Furthermore, with increasing globalization, the international trade market has flourished, and food grains and related products are being traded across multiple countries, requiring intensive tracking. In supply chain management (SCM), the traceability of agricultural products and crops requires robust information management, communications, and the collection of data related to products (e.g., origins and crop exchange information) over their entire life cycle, which can be challenging. The Public Health Organization has observed that product traceability is an essential policy tool for food quality management and monitoring. Dabbene and Gay [2] emphasized enabling product traceability through various tools such as RFID and Bar Codes [3]. In SCM, RFID [4] technology has been employed to facilitate tracking, reduce food wastage, increase operational efficiency, collect data, control aspects such as temperature and humidity, and prevent risks related to shipping and picking.

Cloud computing is now being used for storing information such as the details of food products, customers, and retailers, which can be accessed by users from various websites or barcode scans using mobile phones or other gadgets [5]. Cloud computing also provides instant or small messaging services relating to agricultural products, such as government subsidies, alerts of disease outbreaks, weather conditions, and pesticide information [6]. Cloud computing can be applied at the granular level in the process of keeping food safe by providing the opportunity to analyze and observe the product status from source to destination, i.e., end-to-end delivery. The use of cloud computing and big data is revolutionizing the entire food industry; the cloud structure provides the backbone to analyze and collect data throughout the food supply chain, right from the fields where the crops grow, the warehouses where food is stored, the containers that ship it, to the consumer. They provide a possible alternative to expensive investments relating to hardware and software, allowing the industry to react faster to shifting environments in the marketplace and to gain a competitive advantage. Currently, traceability in the agriculture supply chain suffers from data fragmentation and centralized controls, which cause challenges in data modification and management. Identifying the source and swiftly isolating the product from the supply chain requires close coordination among multiple stakeholders. Individual stages in food supply chains often have good traceability, but the exchange of data and information between stages proves to be difficult to capture and time-consuming.

Many researchers have presented integrations of the food supply chain and blockchain with emerging technologies such as the Internet of things, cloud computing [6], big data, analysis through case studies [7], and survey techniques [8]. These approaches resulted in certain enhancements such as improving traceability efficiency and enhancing transparency in supply chains among users, as well as certain challenges including scalability, immature technologies, lack of legislation, and so on [9]. Blockchain technology is considered a digital ledger that is distributed and organized by a network of various computing devices and machines. A blockchain preserves essential data or information in small chunks called blocks that are secure and cryptographically immutable. Blockchain was first reported in 2008 and is the brainchild of Satoshi Nakamoto. Specifically, the concept of decentralization of the peer-to-peer ledger in 2008 was introduced by Nakamoto. Blockchain technology allows users and suppliers to check transaction details in a real-time environment, as it is used to collect data relating to all transactions occurring within a specific period. Thus, blockchain creates a digital footprint for the verification and validation of data and information [10]. Each blockchain contains several blocks, and each block contains information about the succeeding block in chronological order, as well as the hash of the previous block. Blockchain technology is associated with artificial intelligence (AI) technologies that resolve issues relating to trust, traceability, security, and collaboration in SCM. It is employed in various applications, such as VeChain for certification, Walton chain for apparel supply chains, Ambrosus for food and medicine supply chains, and Modem, exclusively for pharma supply chains. Blockchain technology has been used in the financial domain as the foundation of fully distributed cryptocurrencies such as Bitcoin and in peer-to-peer (or point-to-point) electronic cash systems. It has also aroused interest in various other areas including the food supply chain, medicine, education, e-commerce, real estate, voting systems, and so on.

### 1.1. Blockchain-Related Components

**Figure 1:**
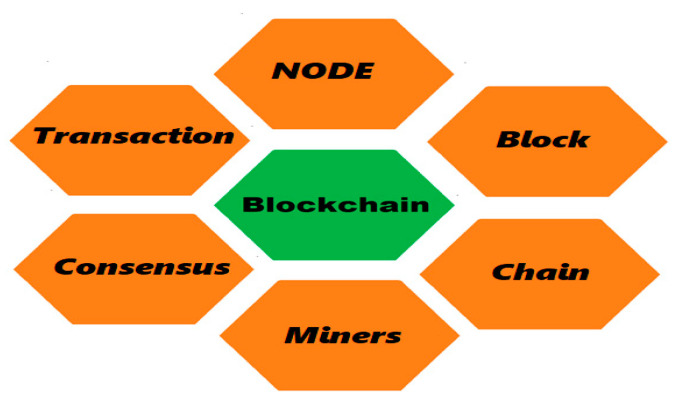
Components of Blockchain.

Given below are the main Blockchain Architecture components also shown pictorically in Figure 1:

- **User or node**—end user or node within the blockchain.
- **Transactions (deals)**—smallest chunks or building blocks for the blockchain transaction system.
- **Blocks or chunks**—a data structure used for preserving a set of transactions, which is distributed to all nodes in the present network.
- **Purchase Chain**—a sequence of blocks related to a purchase order.
- **Miners**—a specific type of user who performs the block verification and validation processes.
- **Consensus**—blockchain operations carried out after verification according to the set of rules and arrangements.

### 1.2. Features of Blockchain

- **Immutability:** Blockchain technology provides the essential benefit that once a user enters information or data into the blockchain, it cannot be updated or modified during the entire transaction process. This characteristic is called immutability, and it has made blockchain technology very popular. Consequently, blockchain is being used in all sectors where data integrity, data security, and data protection are of utmost importance.
- **Autonomy:** Blockchain provides the ability to take decisions individually without intervention by others. It allows the manufacturing and delivery of devices smartly, with IoT-based devices for quick and autonomous decisions in transactions.
- **Decentralization:** All transactions of authorized users can be completed over the internet and accessed without any previous intervention. Every registered user has the same ability to monitor and observe the transaction and prepare copies of all transactions [11]. This information will never be changed without other users being intimated [12]. In contemporary internet-based systems, the entire information of a transaction is not saved only on one single server; copies of the transaction data are saved in distributed computers that are considered “nodes” in the blockchain without any supervisory central authority. Then, all the computers are connected to the blockchain network, which is called a distributed ledger because of the distributed data.
- **Smart Contracts:** A smart contract works as a digitalized contract, and after certain agreements, it operates automatically [13]. In actual fact, a smart contract is a computerized transaction protocol that enhances trust and speeds up transactions [14,15]. For example, once a product is developed and received at the warehouse, payment is made automatically. Using smart contracts, developers can reduce processing time, manpower, paperwork, and other resources. In a new observation, Maersk observed that more than 30 people and organizations were involved in the shipping of containers containing roses, avocados, and other perishable goods from Kenya to the Netherlands in the year 2014 [16]. The entire task was managed using smart contracts only; no human intervention was required, and the entire process took ten days. Smart contracts cannot be changed by humans; they are based on the agreements between partners. Blockchain reduces the risk of transactions at all levels and increases the supply chain visibility, reliability, and transparency while protecting stakeholders’ benefits.
- **Transparency:** Blockchain technology provides a clear and transparent environment. No third party is required as a mediator to provide trust between different parties related to data transactions. Furthermore, even the identities of those involved are hidden with the help of a complex cryptography technique.

This review covers aspects such as how blockchain has been used in the food supply chain and how it can help to address food security and humidity-effect issues. The following questions have been considered:

Question 1: What studies have been conducted on the blockchain with IoT adoption in food supply chain management (FSCM)?
Question 2: What are the benefits of using blockchain in the food supply chain?
Question 3: What are the various challenges of blockchain adoption in FSCM?
Question 4: How does blockchain provide control over FSCM?

The above questions have been answered by collating related papers and summarizing an analysis of the literature. These papers apply a content-analysis-based literature review methodology. Here, we introduce a literature review to provide some background information about the key concepts followed by our research methodology. We then present and discuss the findings, and finally, conclude and discuss future research directions.

## 2. Literature Review

Many researchers have discussed the application of blockchain technology to the food supply chain (FSC). Wang et al. state that FSCs provide a way to design, manage, develop, transition, and systematically organize the food system. Bosona and Gebresenbet [17] explored the notion of food traceability with regard to collecting, storing, transmitting, and preserving food product information throughout the various levels of the FSC using blockchain technology. This further provides control over food quantity, quality, and safety in an FSC. Shih et al [18] described how trading partners for an FSC could preserve a record relating to food transactions. All transactions would be controlled by the trading partners. Lin et al. [19] introduced a traceability system relating to food safety and security, which is based on blockchain technology and a GS1 global standard enabling interoperability (EPCIS). It is specifically used for the process of acquisition, management, and the exchange of product information over the internet. With the help of this system, customers can trace food information through the consumer traceability client application. He et al. [20] developed a nonreversible and decentralized data storage approach related to food. Alonso et al. [21] introduced a unique platform that joins artificial intelligence with IoT, using blockchain technology and edge computing for the management of farming. After using blockchain technology, maintaining food traceability from one end to the other and fragmentation are no longer a challenge. It creates and generates a common platform for data collection. In blockchain technology, consumers can rapidly trace the food forward as well as backward using all the blocks related to products. In another research, “a set of interdependent companies that work closely together to manage the flow of goods and services along the value-added chain of agricultural and food products, to realize superior customer value at the lowest possible costs” was studied. We start the comparison with other companies, then find food products such as producing, by Folkerts and Ko horse [22].

Today, FSCs are centralized and rely on central powers for handling the data and information flow related to food. Several studies have commented on the lack of continuous observation of the FSC and its inability to predict the freshness of the food [23]. Similarly, the conventional food supervision system suffers from various factors, such as inconsistencies in data, interoperability with insufficient resources, fragmentation, and lack of transparency. To mitigate the above concerns, FSC practitioners have adopted various applications related to blockchain technology in the food industry. Near-infrared spectroscopy can be used as a speedy scheme for the evaluation of physicochemical changes in stored foods such as soybeans [24]. Near-infrared spectroscopy (NIRS) is an effective approach for the chemical characterization and screening of agricultural crops. David et al. [25] depict the importance of blockchain technology feasibility in FSCM. With the help of this, organizations can achieve integrity among connected nodes, such as proof of work maintenance, needs, traceability, innovation to reduce intermediaries, and so on. The papers mention a literature review related to sustainable supply chain management (SSCM) that includes the critical factors and performance for developing a comprehensive model of sustainable supply chain management (SSCM) in the food supply chain management industry [26,27]. This paper elaborates on the state-of-the-art relating to blockchain and its applications in supply chain financing and trading. It emphasizes the areas of the blockchain where it may identify the value of trading and supply chain finance [28]. Blockchain technology is a highly growing field in supply chain management, cyber security, banking, and healthcare [29].

Based upon the literature survey, we have identified the few parameters which have been discussed by various papers and it is shown in Table 1. Blockchain is impacting upcoming supply chain practices as well as policies by providing visibility and traceability. Blockchain has the potential to improve as well as enhance the traditional supply chain processes after imposing its own rules, and governance mechanism. In another paper [30], the author discussed how blockchain and IoT-based systems promote value transfer in smart and small-scale agricultural farms. Authors in [31] introduced a method to increase transparency and automate the use of blockchains in agriculture. In another paper [32], the author introduced the various challenges in the implementation of blockchains in the dairy industry. In another paper [33], the author discussed traceability and, with the help of Hyperledger, improved the traceability of a blockchain.

The estimated acceleration in the population of the world and the associated requirement of food from fields to market has also now been sensed with the Internet of underground things (IOUT) [53], by using sensor devices and IoT techniques. The challenge of merging such technologies also requires smart communication between these devices [54]. Due to the fast evolution of IoT, low-power wide-area technologies are becoming popular due to the power concern of these devices. Material conscious information networks (MCIN) are the newly developing techniques that define smart agriculture architecture and also deal with concerns such as management and commerce which affect the supply chain system [55].

## 3. Traditional Supply Chain Management

Traditional supply chain management is very simple and smooth because of clear requirements in terms of design, plans, manufacture, and delivery. Traditional supply chain management (TSCM) is the best practice for the synchronization of the flow of importing and transferring raw materials from supplier to consumer. There is a route from supplier to consumer, e.g., supplier to manufacturer to wholesaler to retailer to consumer. This complete route involves the following stages: generation of order, order collection, gathering information, and timely distribution of goods and services to the consumer. There are 5 V’s (volatility, volume, velocity, visibility, and veracity) relating to SCM to improve the outcomes with several objectives relating to aspects such as services, support, total expenditure, and so on. These objectives can be achieved by the supply manager after applying new digital techniques in the enhancement of supply chain technologies. Furthermore, supply chain managers create additional sources of revenue by providing new access to markets for the creation of smart products. The main function of supply chain centers are movements related to the transactions of raw materials, capital, and finished goods from one place to another. However, the traditional supply chain has the following limitations:

- Traditional supply chains have a limited view of work.
- Delays and unsynchronized responses because of variations in planning.
- Delayed information while passing through each organization.
- The entire chain has limited visibility.
- As information flows, the end customer demands distortion.

Supply chain management involves maintaining information relating to transactions such as the exchange of money, time, and physical materials. An FSC involves various processes which take food from the farm to the dinner table. This includes multiple stages such as the manufacturing, administration, utilization, supplying, and discarding of food products. Food products travel from manufacturers to consumers via workers who work in various stages of the supply chain. At every level of supply chain operations, man-made resources are required to pass the food item toward its destination. Thus, it is essential to streamline the entire supply chain process to prevent high costs, discrepancies, or inefficiencies. The six stages of the food supply chain include:

- **Seed purchasing:** Various food seeds are purchased from seed companies for sale to farmers.
- **Farming**: Ingredients, fruits, meat, vegetables, and beverages originate and are purchased.
- **Processing**: Plants and animals are converted into edible forms.
- **Distributing:** Retailers and suppliers purchase the food in its final form and further transport it. Distributors sell items, manage inventories, reduce costs, and maintain ledgers to give value to food products.
- **Retailing**: The food product is delivered to the final consumers.
- **Food product purchasing**: The final stage of FSC where the consumer purchases the finished product from the retailer.

**Figure 2:**
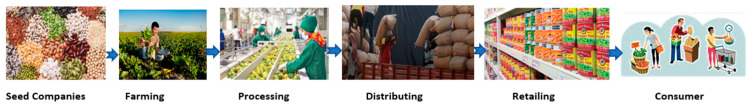
Supply chain management.

In the Figure 2, a simple supply chain is shown. Seed companies will provide raw materials like seeds, plants, and so on. Farmers [56] purchase raw materials and use them for cultivation. After harvesting, the foodstuffs will further move toward processing. During processing, factories process foodstuffs and forward the useful portions for distribution in the next phase. Distributors sell this food to retailers, and customers then purchase items from retailers.

## 4. Blockchain with IoT Devices

The Internet of things (IoT) is an intelligent, reliable, and high-speed information network that connects objects for data collection and transmission. IoT includes radio-frequency identification (RFID), a global positioning system (GPS), a geographic information system (GIS), a wireless sensor network (WSN), and so on. IoT provides the facility of automatic recording through IoT sensors which collect information such as temperature, voice, and humidity. In SCM, IoT provides real-time data collection for fresh food products, which is used to identify the quality of the product concerning the external environment [57]. IoT devices help to eradicate human error and increase the efficiency of monitoring and capturing information. IoT in conjunction with blockchain enables smooth transactions and the monitoring of data transfer through blocks. There are several emerging areas of IoT with blockchain.

### 4.1. IoT for Healthcare

In recent years, healthcare services have undergone drastic changes in response to increased demand. There are several wearable devices [46] that can identify the medical state of patients. Wearable devices can determine a patient’s blood pressure, blood glucose, breathing problems, heartbeat, and so on [58]. However, while wearable devices can be used to transfer and collect data only in hospitals, IoT devices must be used to monitor patients remotely, for which remote monitoring systems are needed. Some of the devices used for healthcare monitoring are:

- Stationary medical devices;
- Medical embedded devices;
- Medical wearable devices;
- Wearable health monitoring devices;
- Glucose monitoring devices;
- Hand hygiene monitoring devices;
- Depression and mood monitoring.

Stationary medical devices are physically installed in specific locations. Embedded devices are placed inside the patient’s body. Medical wearable devices, prescribed by doctors, and wearable health monitoring devices are used to monitor the health of the patient and then relay that information to the concerned persons. Blockchain technology works with IoT-enabled systems to maintain the medical records of patients. A blockchain [39] maintains the ledger of data relating to a patient, and their doctor can use this ledger to extract the data.

### 4.2. IoT for Smart Homes

Smart Homes provide an automated and intelligent system to protect homes from outsiders. Through the Internet of things (IoT), smart electronic devices such as Smart TVs, LED lights, microwaves, AC, and refrigerators can be connected through the internet, and humans can control various household activities. IoT-based smart homes feature a one-to-one communication system between various devices without human intervention. In IoT-based smart homes, the gateway for communication can be configured using blockchain technology which can be used to store and exchange data in the form of chunks or blocks. Blockchain technology [41] also provides decentralization support to overcome issues arising in traditional centralized architecture.

**Figure 3:**
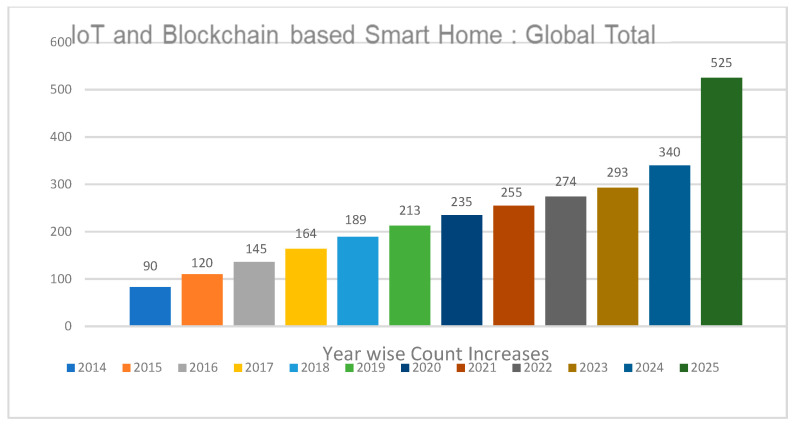
IoT and Blockchain-based Smart Homes.

The number of smart homes is increasing every year, and the price for the development of IoT-based networks is also increasing. Surveys claim that worldwide consumer spending in the smart home sector added up to nearly $90 billion in 2014 and $213 billion in 2019, and it is expected to grow at 10% CAGR to $525 billion over the forecasted period from 2019 to 2025 as can be seen in Table 2. Figure 3 shows the year-wise increase in the count of the smart IoT and blockchain based smart devices.

As an example, in smart homes, we might have a smart refrigerator that may indicate the status of the left-over food in the refrigerator, such as a small egg tray, and may send the status signal of left-over eggs to the owner or to the shopkeeper from where the materials are normally purchased. Similarly, a milk bottle may indicate the amount of milk left in the bottle to the shopkeeper for the supply of another bottle(s). As another example, the smart storage food tray may indicate the freshness status of vegetables stored in it and this way items may be supplied directly to the home.

### 4.3. IoT for Supply Chain Management

At present, SCM [31,34] is an emerging area of blockchain technology. SCM can be effective if it provides reliable visibility into materials and goods, from the production level to the delivery of the product. IoT has enabled manufacturing companies to achieve such visibility by using smart SCM which is IoT-enabled to optimize production as well as good transmission. Smart SCM is improving the customer delivery service as well. In such systems, multiple IoT-enabled sensors work in various stages of the supply chain to provide functionalities such as real-time monitoring throughout the supply chain, which can be used to monitor raw goods and materials quality. These sensors identify and respond according to real-time changes happening in an environment. Sensors receive inputs from a variety of sources such as light, temperature, motion, and pressure. Sensor technology has made it easy for businesses to transition to IoT-enabled supply chains:

- **Customer satisfaction as per requirements**: At the time of manufacturing, companies identify food that requires special arrangements for shipping and transport. Smart SCM maintains regulatory compliance and high quality throughout the shipment transport.
- **Safeguarding:** Smart IoT food supply chain monitoring systems offer quality identification, consumer assurance, and food validation after meeting customer expectations.
- **Cost-effectiveness**: Smart SCM provides a cost-effective IoT-based system to identify the food quality and shipment from manufacturing to delivery.
- **Maintaining Integrity:** While manufacturing and distributing, certain foods, products, and pharmaceuticals require special preservation to prevent harm. An IoT-based SCM works to maintain the integrity of perishable food. Smart supply chains preserve human health and eliminate waste through stringent monitoring and alerts.
- **Trackability:** IoT devices trace the food freshness level at every transaction point. These collect the food condition information and transmit it to the cloud for further storage. The blockchain collects this information and maintains a ledger for the particular food. Consequently, the transporter and seller can check the condition of the food at every stage.

**Figure 4:**
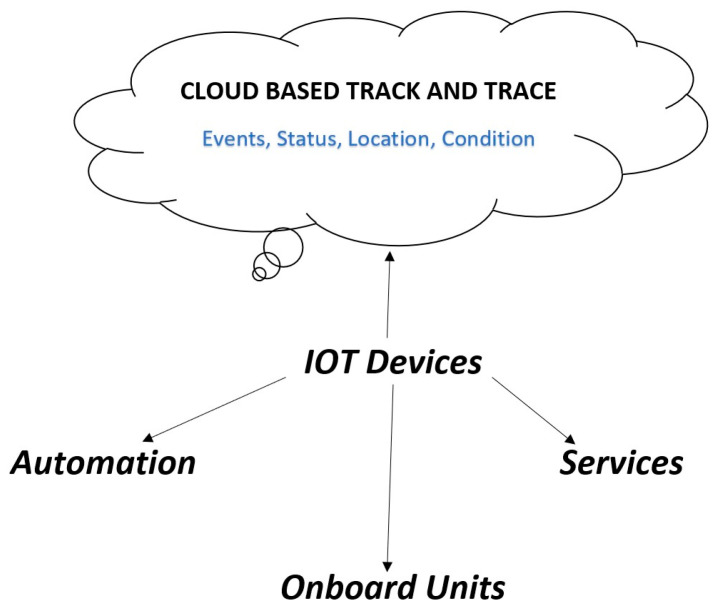
SCM based on IoT and blockchain.

In Figure 4 shown that modern supply chains have numerous entities, such as devices, automation, transportation, onboard units, services, and so on, with proper components and capacity. Each level of the SCM has been identified and coordinated, and transparency between them is provided. Traditional supply chains work independently at each stage: manufacturing, marketing, distribution, planning, and finally, organizational purchasing. However, individual components of supply chains can be directly connected using IoT devices. IoT devices receive data or information from sensors installed at various stages in the supply chain. This data is then transmitted to a cloud [29] for permanent storage, as shown in the above figure. Researchers observed that combining blockchain technology with IoT effectively provides scalability, security, efficiency, auditing, quality, and interoperability. A self-organized blockchain and IoT-based agriculture system that worked without human intervention was introduced by Lin et al. [47]. In IoT and blockchain environments, tracking initializes from business partners to the end consumer. This whole task is achieved through cloud-based technology where various aspects such as events, status, location, and conditions are monitored. Then, status updates are stored in the status table for further perusal. The location and condition of an event can also be tracked by the cloud throughout the business onboarding, to consumer purchasing and receiving. IoT devices include sensors that record conditions such as temperature and humidity. The collected information is stored in a cloud that is remote and secure. Cloud-based technology associated with blockchain and IoT performs various operations such as maintaining food pedigree and safety, multi-tier logistics network visibility, supply chain integrity, real-time inventory management, real-time planning predictions, and so on. The blockchain creates ledgers for the data and maintains a record of food. However, blockchain technology has certain challenges, e.g., there is no way to change or update data in a blockchain if mistakes occur. It is difficult to manipulate and amend data or information in blockchains. Various security transaction schemes do exist but are difficult to verify. Installed IoT devices can be hacked by hackers.

## 5. Conclusions

A brief literature review is essential to provide a quick analysis of the published research. This review can help researchers determine future directions in developing applications involving IoT with blockchain technology in FSCM. Researchers can observe the challenges and potentials of blockchain in the FSC. In this paper, 60 research papers (journal articles, conference papers, and book chapters) relating to blockchain and IoT have been reviewed and some observations have been presented. Applications of IoT and blockchain in other fields, such as healthcare and smart homes, have also been presented. Blockchain and smart contracts are used to track and trace the performance of business transactions after eliminating intermediaries. At each stage of transactions, IoT devices observe and record the food condition. This information is further stored on the cloud and the blockchain creates a ledger. Blockchains have improved food traceability and enhanced FSC trading activities through collaborative relationships and by maximizing operational efficiencies. However, the study findings are that the blockchain falls under four basic categories: Member name, technical issue, organizational effect, and regulatory barriers. These all include some issues, such as scalability, security, and privacy in the blockchain. This paper shows how the blockchain with IoT influences the food supply chain and the fundamental concepts, and the understanding of blockchain with IoT technology. It is a guide toward the new and relevant research areas of blockchain concerning the food supply chain. This paper includes the technological adoption of blockchain as well as the adoption of challenges for the food chain with IoT devices.

## Figures and Tables

**Table 1:** Literature review for the supply chain.

Research Paper	Customer Intervention	Implementation System	Froud Detection	Traceability	Price Transparency	Original Database	Based on IoT
[33]	**√**			**√**	**√**	**√**	
[34]		**√**	**√**	**√**	**√**	**√**	**√**
[35]	**√**			**√**		**√**	**√**
[36]		**√**	**√**	**√**			
[37]					**√**		**√**
[38]	**√**		**√**	**√**		**√**	
[39]			**√**	**√**		**√**	**√**
[40]	**√**		**√**	**√**		**√**	**√**
[41]	**√**	**√**		**√**			
[42]					**√**		**√**
[43]		**√**	**√**			**√**	
[44]				**√**			
[45]	**√**	**√**	**√**	**√**			
[46]	**√**				**√**		
[47]				**√**		**√**	**√**
[48]	**√**	**√**		**√**		**√**	
[49]	**√**			**√**			**√**
[50]	**√**	**√**					**√**
[1]	**√**		**√**	**√**		**√**	**√**
[51]	**√**			**√**		**√**	
[52]		**√**		**√**		**√**	
[25]	**√**			**√**		**√**	

**Table 2:** Annual count increases and annual spending.

Years	Annual Increase	Annual Spending Per Count
2014	90	$48
2015	120	$60
2016	145	$72
2017	164	$80
2018	189	$96
2019	213	$108
2020	235	$121
2021	255	$132
2022	274	$143
2023	293	$155
2024	340	$167
2025	525	$201

## Data Availability

Not applicable.
